# P-952. A Proposed National Quality Improvement Curriculum for Pediatric Infectious Diseases Trainees

**DOI:** 10.1093/ofid/ofae631.1142

**Published:** 2025-01-29

**Authors:** Caitlin Li, Hannah Alkema

**Affiliations:** Lurie Childrens Hospital, Chicago, Illinois; Ann & Robert H Lurie Children's Hospital of Chicago, Chicago, Illinois

## Abstract

**Background:**

Quality improvement is a required competency for all pediatric fellowships, and this skill set is particularly important for infectious diseases (ID) clinicians. However, almost half of pediatric ID program directors report that they do not have a formal QI curriculum for their fellows.

Proposed Quality Improvement and Patient Safety Framework

**Figure:**
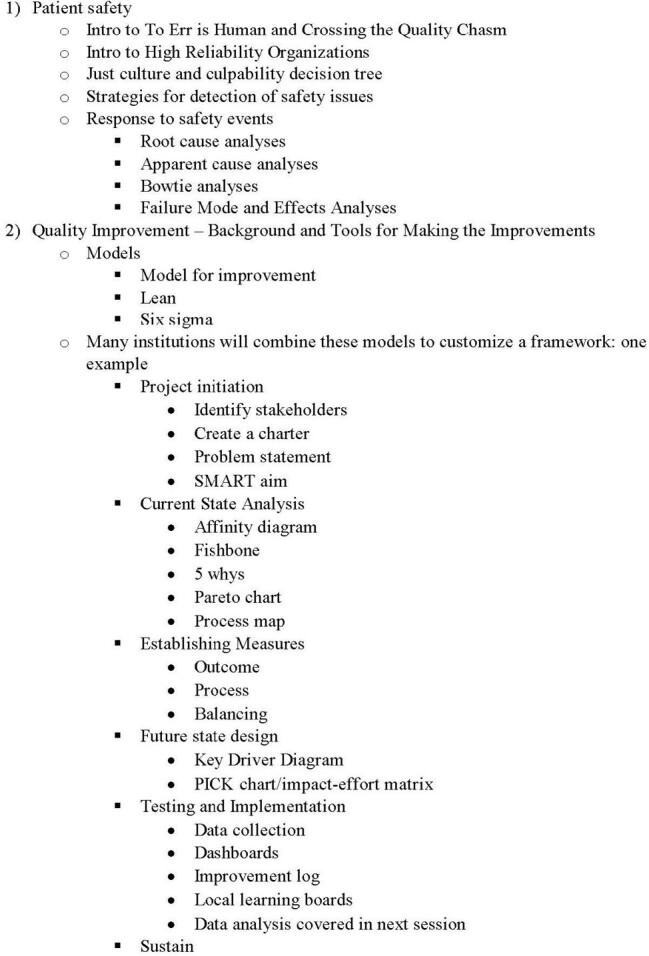

**Methods:**

A pediatric infectious diseases attending with fellowship and Master’s level training in QI collaborated with the director of improvement science at a large, standalone pediatric hospital to adapt a successful local QI workshop curriculum to align with the needs of pediatric ID fellows. Pediatric ID programs of varied sizes and geographic locations were identified as test sites so the program could be iteratively improved prior to scaling to the national level.

**Figure:**
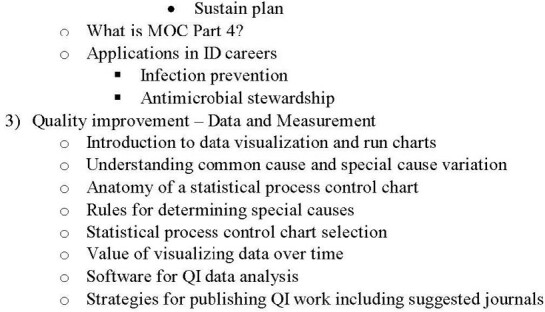

**Results:**

In current state, the designed curriculum has three sections: Patient Safety, Quality Improvement – Background and Tools, and Quality Improvement – Data and Measurement. Each section is designed to be available as a recorded lecture that can be viewed asynchronously or a set of slides with lecture notes that can be reviewed by the participant at their preferred pace. The curriculum will be presented to pediatric fellows at identified programs and iterated based on their feedback prior to widespread distribution.

**Conclusion:**

A national QI curriculum for pediatric ID fellows fills a current gap in resources available for training the next generation of pediatric ID physicians and will equip them with tools that are critical for many facets of a career in ID.

**Disclosures:**

**All Authors**: No reported disclosures

